# Rare exclusive hadronic *W* decays in a $t\bar{t}$ environment

**DOI:** 10.1140/epjc/s10052-015-3482-x

**Published:** 2015-06-11

**Authors:** Michelangelo Mangano, Tom Melia

**Affiliations:** TH Group, PH Department, CERN, 1211 Geneva 23, Switzerland

## Abstract

The large cross section for $t\bar{t}$ production at the large hadron collider (LHC) and at any future hadron collider provides a high-statistics and relatively clean environment for a study of *W* boson properties: after tagging on a leptonic decay of one of the *W*s and the two *b* jets, an additional *W* still remains in the event. We study the prospect of making the first exclusive hadronic decay of a fundamental boson of the standard model, using the decay modes $W\rightarrow \pi \gamma$ and $W \rightarrow \pi \pi \pi$, and other related decays. By using strong isolation criteria, which we impose by searching for jets with a single particle constituent, we show that the three-particle hadronic *W* decays have potential to be measured at the LHC. The possibility of measuring an involved spectrum of decay products could considerably expand our knowledge of how the *W* decays, and experimental techniques acquired in making these measurements would be useful for application to future measurements of exclusive hadronic Higgs boson decays.

## Introduction

Experimental measurements of the decay modes of the *W* boson are summarised in the Particle Data Group (PDG) review [1]. They consist of: the three leptonic decays to $e,\mu ,$ and $\tau$ plus a neutrino (with $\sim$1 % precision), and the total decay rate to leptons; inclusive hadronic decay ($\sim$0.5 % precision), which is split further into inclusive hadronic decays to *cX*, and $c\bar{s}$; and invisible decay (consistent with zero at a level of $\sim$3 %). In addition to this, two 95 % confidence level upper limits are set on the decay rate, $\Gamma _i$, for $W^+\rightarrow \pi ^+\gamma$ ($\Gamma _{\pi \gamma }/ \Gamma _{\text {tot}} < 8 \times 10^{-5}$)^1^ and $W^+\rightarrow D_s^+\gamma$ ($\Gamma _{D_s\gamma }/ \Gamma _{\text {tot}} < 1.3 \times 10^{-3}$), making up a total of ten entries in the table.

This is to be compared with the PDG table for the *Z* boson, which reports over 50 different searches and measurements of the decay modes of this particle, including semi-exclusive hadronic final states (e.g. $$Z\rightarrow J/\psi \,X$$, $$D^\pm \, X$$, $$B^0_s\, X$$, etc.), as well as upper limits on fully exclusive hadronic decays (e.g. $$Z\rightarrow \pi ^0\gamma $$, $$\pi ^\pm W^\mp $$) and on lepton flavour, lepton number and baryon number violating decays (e.g. $$Z\rightarrow \mu e$$, *ep*). The leptonic decays and the total inclusive hadronic decay of the *Z* have been measured with a precision over an order of magnitude better than those of the *W*, i.e. at the per mille level. The difference in the PDG tables reflects the fact that LEP, being an electron–positron collider, could singly produce of order $$10^7$$*Z* bosons in an experimentally clean environment. Although $$\sim $$$$10^{11}$$*W* bosons will be produced at the high luminosity (HL) large hadron collider (LHC), and orders of magnitude more at proposed future hadron colliders, the huge QCD background to generic *W*-production final states, and the trigger challenges, render many precision studies of *W* decays implausible at these machines. Proposed future electron–positron colliders will pair produce *W* bosons in a clean environment, but, even in the case of circular accelerators such as TLEP [3] or CEPC [4], they at best promise samples of $$\sim $$$$10^8$$ events. Can the hadron collider experimental barrier be overcome and the huge statistics be exploited?

**Fig. 1:**
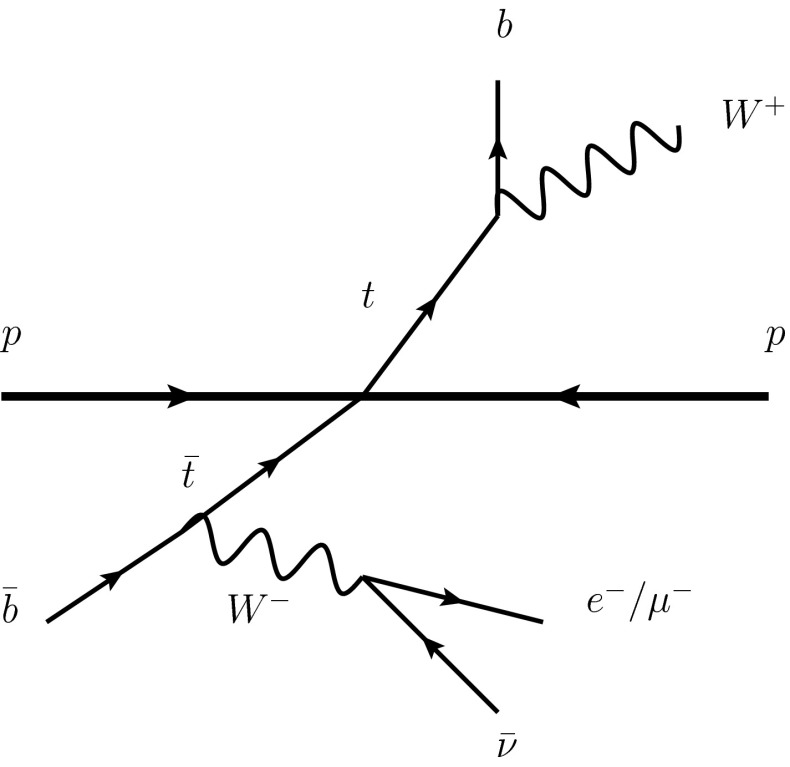
The $t\bar{t}$ environment in which the *b*-jets are tagged on, and the leptonic decay of one of the *W*s is required

One of the main ideas in this note is to highlight that the enormous $t\bar{t}$ production cross section at hadron colliders operating at LHC energies and above is a promising environment in which to make precision measurements of the *W* boson, given the manageable QCD background and given the trigger opportunities. Top quarks decay dominantly into a *b* quark and a *W*, and by tagging on the leptonic decay of one of the *W* bosons in the event, as well the *b*-jets, a situation is created where inclusive decays of the leftover *W* boson in the event can be studied in a rather unbiased way – see Fig. 1. There will be $O(10^{9})$*W* bosons potentially triggerable in this way at the end of the HL-LHC run, and $O(10^{11})$*W*s at a 100 TeV collider taking 10ab$^{-1}$ of data, i.e. orders of magnitude more than *Z* bosons at LEP or *W* bosons at a future circular $e^+e^-$ collider. This possibly opens a door to a high-statistics program of *W* boson studies, including searches for rare or forbidden decays (e.g. lepton flavour and lepton number violating decays [5]), and improved measurements of leptonic and hadronic branching ratios.

In this note we focus on fully exclusive hadronic decays of the *W*, which are experimentally very difficult to study at a hadron collider. For this we use a technique that utilises what can be seen as an extreme form of jet substructure and which we refer to as single particle jet isolation – requiring jets which have as constituents a single particle (with a looser definition when the particle is a photon). This method relies on the fact that a well-isolated single hadron or photon is a rare outcome of generic QCD evolution. This approach is clearly analogous to what is done experimentally to identify hadronic decays of tau leptons.

Of the three massive fundamental bosons of the standard model, not a single exclusive hadronic decay mode has ever been measured. Low-multiplicity decays can only arise from a perturbative evolution of the final state with radiation of few (or no) gluons, with a probability that is greatly suppressed by Sudakov effects in the form of powers of $\Lambda _{\mathrm{QCD}}/m_W$. The observation of such decays would therefore probe strong-interactions in a very interesting dynamical domain, at the borderline of perturbative and non-perturbative physics. Furthermore, a number of recent papers [6–11] have addressed the idea of using exclusive hadronic decays of the Higgs boson $h\rightarrow VM$, where $V=W,Z,\gamma$ and *M* is a meson, as a test of both the on- and the off-diagonal couplings of *h* to quarks. Such measurements are very challenging at the LHC, and observing exclusive hadronic decays of the *W* would provide a proof of principle that this type of final state is accessible at a hadron collider. As pointed out in [8], future electron–positron colliders do not have the required statistics for observing these decays.

We present a Monte Carlo (MC) study, performed at particle level, using single particle jets to overcome the overwhelming hadronic activity at a hadron collider. We focus on the phenomenologically simplest two- and three-particle exclusive decays to mesons that are ‘stable’ as far as the LHC detectors are concerned,

$$\begin{aligned}&W^+ \rightarrow \pi ^+ \gamma ,\\&W^+ \rightarrow \pi ^+\pi ^+\pi ^{-}, \end{aligned}$$

and we show that requiring single particle jets provides an excellent handle for separating signal from background. We highlight that three-particle decays in particular are candidates for an LHC measurement. Related decays with pions substituted by other charged particles, such as $K^+$, $D^+_s$ etc. are also discussed, as well as the prospect of mass measurement in these fully visible decay modes.

We proceed as follows: in Sect. 2 we discuss theoretical issues surrounding exclusive hadronic decays of weak bosons. In Sect. 3 we present a MC particle level study which uses the technique of single particle jet isolation to measure these decays at hadron colliders, and comment on further experimental handles that can be used to increase sensitivity to these decays in the $t\bar{t}$ environment. In Sect. 4 we present our conclusions and outlook, including implications of our results for exclusive Higgs boson decay.

## Rare exclusive hadronic decays of the *W* boson

**Fig. 2:**
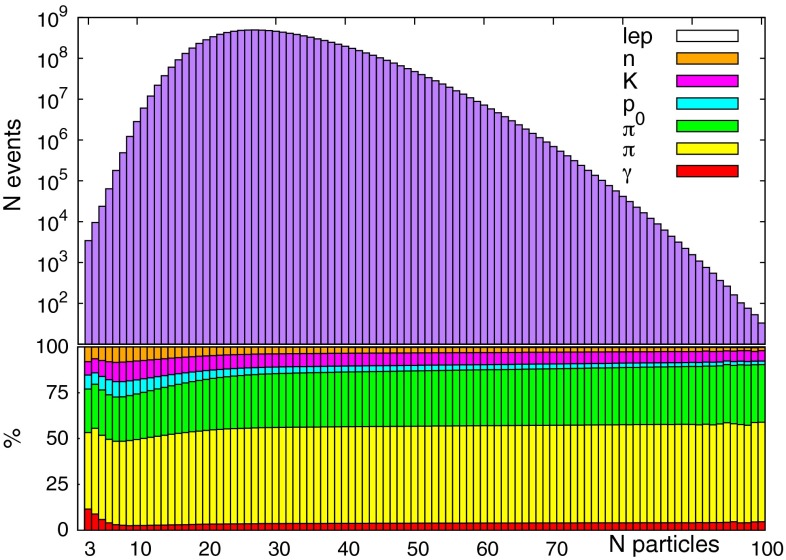
The distribution of the number of final state particles in $10^{10}$ showered and hadronised $W\rightarrow u\bar{d}$ decays in PYTHIA 8. *Top panel* gives total number of events. The legend corresponds to the *bottom panel* where the average fraction of types of particle in the final state are given (in *descending order*): leptons (not visible on *plot*), neutrons, kaons (both charged and neutral), protons, neutral pions, charged pions, and photons

The main reason that no exclusive hadronic final state of the weak bosons, *W* or *Z*, has ever been observed is because the majority of the decays are into $$\sim $$30 particle final states (as seen by the detector), composed of charged and neutral pions and kaons, protons, neutrons, photons, and leptons. To get a feel for the distribution of final states, we show in Fig. 2 the result of decaying, showering and hadronizing $$10^{10}$$$$W^+\rightarrow u\bar{d}$$ with PYTHIA 8 [12, 13], letting any resonances decay to particles seen in the detector (except for neutral pions, which we keep undecayed – these will decay $$\pi ^0\rightarrow \gamma \gamma $$) and counting the number of each type of particle produced. Clearly, a measurement of branching fractions to any of the given exclusive, high-multiplicity final states that dominate the decay is implausible, especially as many of the decay products are neutral and hard to identify. However, PYTHIA does find that a number of three-particle final states are possible and can be found in the MC final state:

$$\begin{aligned}&W^+ \rightarrow \pi ^+ \pi ^+ \pi ^-,\\&W^+ \rightarrow \pi ^+ p \, \bar{p} , \\&W^+ \rightarrow \pi ^+ K^+ K^- ,\\&W^+ \rightarrow \pi ^+ n^0\, \bar{n}^0, \\&W^+ \rightarrow \pi ^+ \pi ^0 \gamma ,\\&\ldots , \end{aligned}$$

some of which contain only charged hadrons. The rate at which these final states occur is one every $$10^6$$–$$10^7$$ events, but the PYTHIA models are constrained by charged multiplicity data from LEP measurements of $$Z\rightarrow $$ hadrons which have large errors in the extremities of the multiplicity distributions (for the most recent LHC PYTHIA tune and a detailed discussion, see [14], and references within). In order to obtain an estimate of the robustness on this PYTHIA-extrapolated branching ratio, we use a variety of different available tunes, including one which does not include multiplicities: the envelope remains between one in $$10^6$$ and $$10^7$$. We expect from the perturbative QCD picture that the decay $$W\rightarrow \pi \pi \pi $$ would have a rate of the same order of magnitude as $$Z\rightarrow \pi ^0 \pi \pi $$. We are not aware of direct searches for such a final state, and cannot infer, from the available information on the study of hadronic final states at LEP, what the constraints on its branching fraction could be. Given $$\sim $$$$ 10^7$$*Z* bosons were produced at LEP, however, no constraint below $$O(10^{-6})$$ can likely be obtained. The value obtained above with PYTHIA is therefore consistent with LEP. The LEP results should be interpreted as an experimental upper limit that the branching ratio is unlikely to exceed $$\sim $$$$ 10^{-5}$$.

**Fig. 3:**
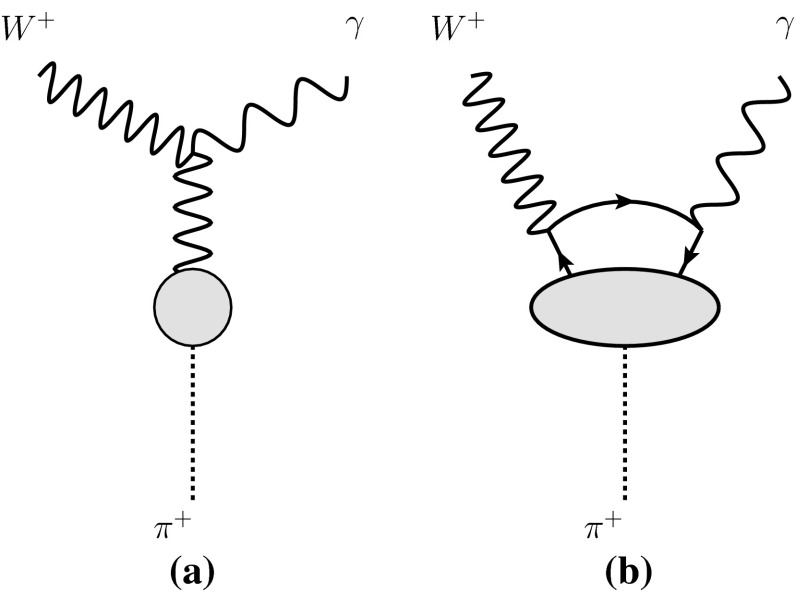
Contributions to the decay $W^+ \rightarrow \pi ^+ \gamma$

The two-particle decay mode $W^+\rightarrow \pi ^+\gamma$ was not found by PYTHIA in the above exercise of showering and hadronizing $W^+\rightarrow u\bar{d}$. This region is very extreme as it requires the *u* and $\bar{d}$ quark to be recoiling against a photon, with a very small invariant mass. What is more, a contribution to the decay where the photon couples directly to the *W* boson, shown in Fig. 3a, is not taken into account. The rate can be estimated as follows. Contribution (a) can be related directly to the pion decay constant, $f_\pi =93\,$MeV, via an evaluation of the current,

1 $$\begin{aligned} \langle \pi ^+(p) | J_W^\rho (0) | 0 \rangle = \frac{f_\pi }{\sqrt{2}}\,\, p^\rho , \end{aligned}$$

where *p* is the momentum of the pion state $|\pi ^+(p)\rangle$, and $J_W^\rho = \bar{d}\gamma ^\rho P_L u$ is the weak current, with $P_L=\frac{1}{2}(1-\gamma _5)$. Contributions of the type shown in Fig. 3b involve a calculation of

2 $$\begin{aligned} \int {\mathrm{d}^4 x e^{i k\cdot x} \langle \pi ^+(p) | T[ J_W^\lambda (0) J_\gamma ^\mu (x)] | 0 \rangle }, \end{aligned}$$

where *k* is the photon momentum, and $J_\gamma ^\mu = \sum _{i=u,d}Q_i\bar{q}_i\gamma ^\mu q_i$ is the electromagnetic current, with $Q_i$ the charge of the quark $q_i$. To evaluate these contributions we adapt Manohar’s calculation of the decay width $Z\rightarrow W^\pm \pi ^\mp$, and subsequent estimate of the decay $Z\rightarrow \pi ^0 \gamma$ [15], which uses an operator product expansion (OPE) at leading order in the strong coupling constant $\alpha _S$, retaining only the leading terms in a tower of twist two operators. We review this calculation in the appendix. We obtain an order of magnitude estimate for $\Gamma _{\pi \gamma }/\Gamma _{\text {tot}}\sim 10^{-9}$, although the expansion is not convergent and will be modified by important higher order corrections.^2^ This result can be compared to previous results in the literature for this decay. A calculation by Arnellos, Marciano and Parsa (AMP) [17] also yields a value for $\Gamma _{\pi \gamma }/\Gamma _{\text {tot}}$ around $10^{-9}$, assuming the Brodsky–Lepage (BL) asymptotic formula [18] for the off-shell photon–photon–pion vertex, $\gamma ^\star \gamma \pi$, for both the vector and the axial form factors.^3^ A one-loop calculation by Keum and Pham [30] gives a prediction of $\Gamma _{\pi \gamma }/\Gamma _{\text {tot}}\sim 10^{-8}$–$10^{-6}$. This calculation follows closely the very similar calculation of the famous $\pi ^0 \rightarrow \gamma \gamma$ anomaly [31, 32] and is obtained via a use of the Goldberger–Treiman relation [33] for the quark–quark–pion vertex, yielding $\Gamma \propto m_q^4$, where $m_q$ is the quark mass. The upper value of $10^{-6}$ follows from using current quark masses $m_q\sim 300\,$MeV in the loop. The Goldberger–Treiman relation is not valid if the momentum running in the quark loop is truly at the scale of the *W* mass, i.e. much greater than $4\pi f_\pi$. Because of this, we expect that the upper estimate of $10^{-6}$ is too large.

Is it possible to observe final states with branching ratios as small as those described above? We now turn to the challenge of observing them in high energy proton–proton collisions, where pions and photons are produced in huge numbers, and study the use of single particle jet isolation in the $t\bar{t}$ environment.

## Single particle jet isolation

We perform a MC study to estimate the reach of the HL-LHC and future hadron colliders in observing such two- and three-particle exclusive hadronic decays, with two separate analyses to search for $W\rightarrow \pi \gamma$ and $W\rightarrow \pi \pi \pi$ as explicit examples. The numbers presented in this section are for 14 TeV *pp* collisions, and we discuss the scale-up to 100 TeV in the following section. We generate $t\bar{t}$ events where one of the *W*s decays as $W^-\rightarrow e^- \bar{\nu }_e$, using MadGraph 5 [34] at tree-level. The two separate signal samples are generated by then forcing the $W^+$ to decay to $\pi ^+\gamma$ or $\pi ^+\pi ^+\pi ^-$ isotropically (neglecting spin effects^4^). A background sample is generated by allowing the $W^+$ to decay generically hadronically – we refer to this as the ‘*W*-had’ background.^5^ We separately generate the background where the $W^+$ decays into a tau lepton, referred to as the ‘*W*-tau’ background, where the tau can then decay hadronically. Since a tau decays to one or three charged pions and kaons $\sim$12 and $\sim$15 % of the time, respectively, this could be an important background. We also considered QCD $W^-b\bar{b}$ production, with $W^-\rightarrow e^- \bar{\nu }_e$, which is an irreducible background to the tagging procedure for the $t\bar{t}$ environment; this background is subdominant to the ones above (as well as displaying a very different event topology to the $t\bar{t}$ one, which could be utilised to suppress it further) and we discuss it no further here. In all samples, the electron from the *W* decay is required to be separated from the *b* partons by $\Delta R_{eb}>0.3$. These samples are then showered and hadronised with PYTHIA 8, without the addition of pileup.

The analysis involves the following steps, which we describe in detail below:

- Select $t\bar{t}$ events by requiring an electron, missing energy, and two *b*-jets constructed with cone size $R=0.4$.
- Remove all particles associated with the *b*-jets from the analysis.
- Re-cluster the remaining particles using a cone size $R=R_{\mathrm{iso}}$ and require a single particle jet (defined below) for each final state hadron of the decay.

For the triggering cuts, designed to select the $t\bar{t}$ event, jets are reconstructed with FastJet [36], using the anti-kT algorithm [37] with $R=0.4$, and requiring the transverse momentum of the jet $p_T^j>25\,$GeV and rapidity $|\eta _j|<2.5$. The constituents of these jets are analysed and a jet is considered *b*-tagged if any of its constituents is a *b*-hadron. We require two *b*-tagged jets. The electron is required to have $p_T^e>20\,$GeV and $|\eta _e|<2.5$, and the missing transverse momentum $p_T^{\text {miss}}>30\,$GeV (constructed as the modulus of the vector sum of the transverse momentum of all final state particles with $|\eta |<3.6$, except for neutrinos). We choose not to impose a transverse mass cut on the top quark. Such a cut would suppress any QCD background, but the background we consider here is found to be suppressed enough by the following selection criteria, so it is advantageous in this analysis to keep as much signal as possible.

If the event passes these triggering cuts, single particle isolation cuts are then implemented to separate signal from background. Firstly, all of the particles associated with the two *b*-tagged jets are removed from the event. The event is then resent to FastJet with a different *R* parameter, $R=R_{\text {iso}}$, which we vary in the following, and we again construct jets with $p_T^j>25\,$GeV, $|\eta _j|<2.5$. We call a jet a “single pion jet” if it is composed of exactly one charged pion. Similarly a “single $\gamma$”-jet is defined when all of the constituents of the jet are photons. This definition is loose in the sense that no differentiation is made between MC jets consisting of a single photon and those containing two or more photons (for example coming from the decay of a $\pi ^0$). We assume for now charge and particle identification used in the definition of the single pion jet. For the $\pi \gamma$ analysis, events pass these selection cuts if at least one $\gamma$-jet and one charged single pion jet are found. For the $\pi \pi \pi$ analysis, we require at least three charged single pion jets. If more single particle jets are found, the ones with the hardest transverse momentum are selected (this happens a negligible amount of the time).

**Fig. 4:**
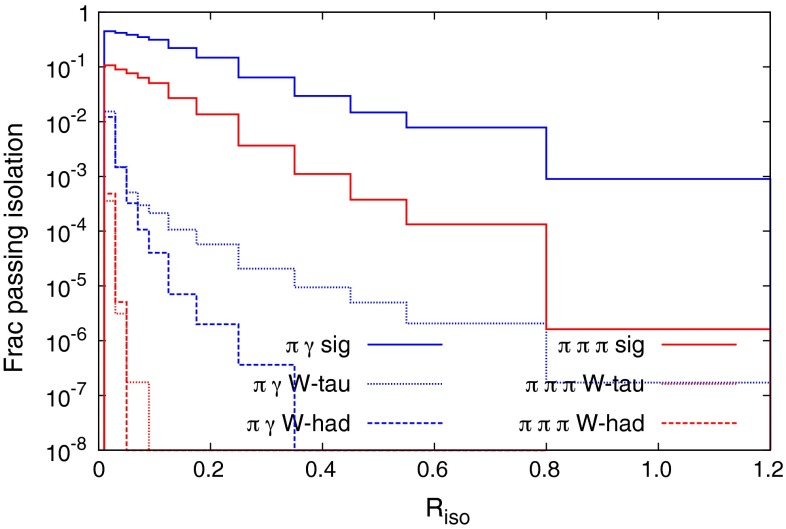
Fraction of signal and backgrounds passing the single particle jet isolation cuts as a function of the parameter $R_{\mathrm{iso}}$

**Fig. 5:**
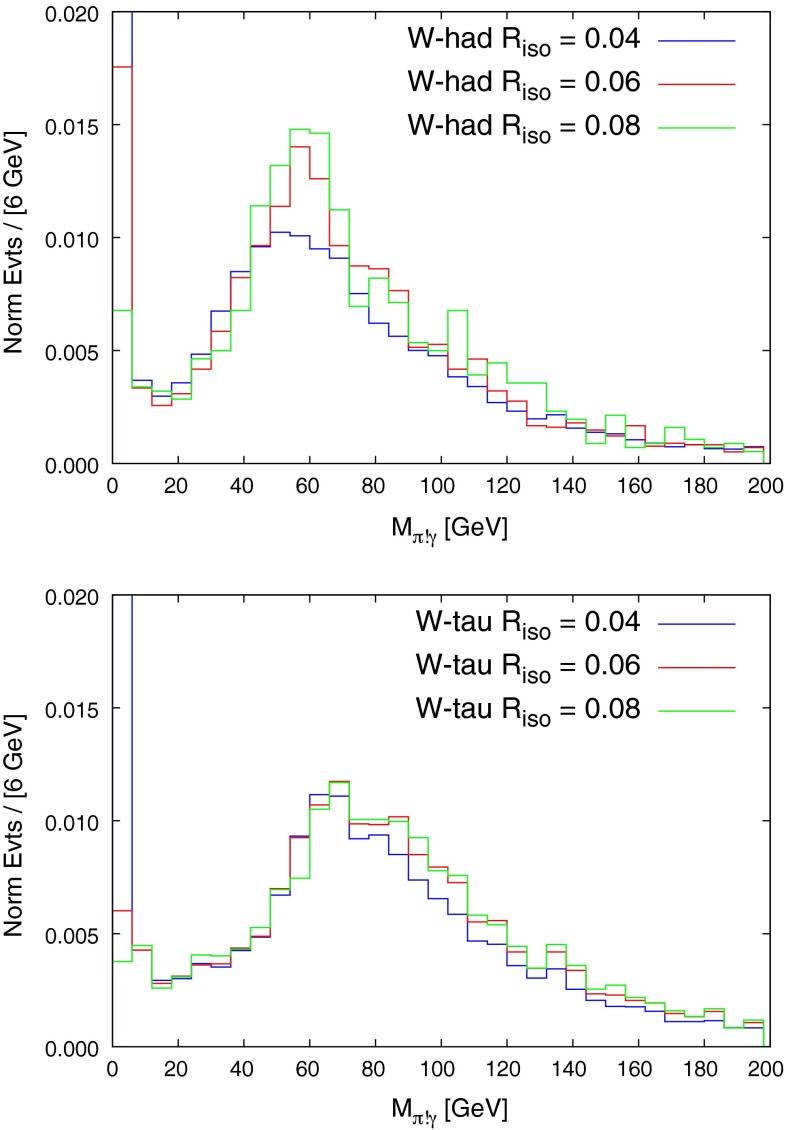
*Top* distribution of $M_{\pi \gamma }$ for the generically hadronically decaying *W* background in the $W\rightarrow \pi \gamma$ analysis, plotted for different values of $R_{\mathrm{iso}}$. *Bottom* distribution of $M_{\pi \gamma }$ for the *W* decaying to a tau-lepton background in the $W\rightarrow \pi \gamma$ analysis, plotted for different values of $R_{\mathrm{iso}}$

**Fig. 6:**
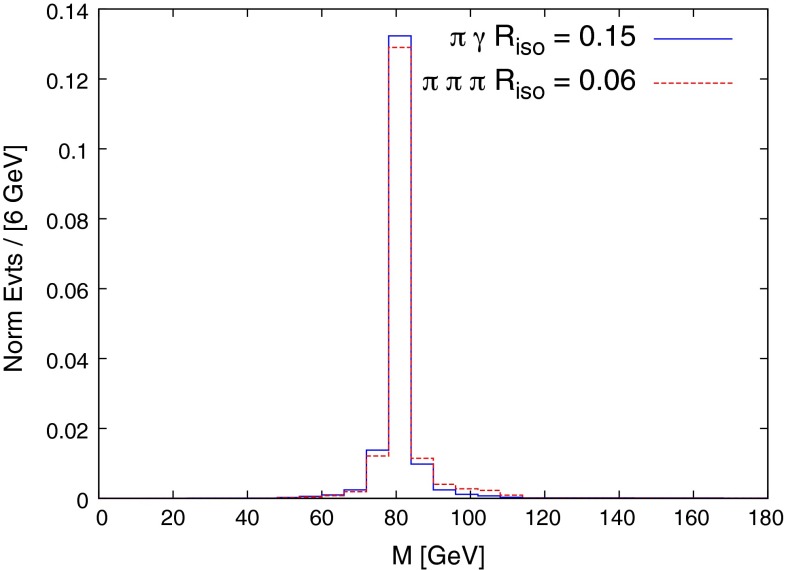
Signal distribution of the reconstructed *W* mass for the decays $W\rightarrow \pi \gamma$ and $W\rightarrow \pi \pi \pi$

The fraction of signal, *W*-had background, and *W*-tau background passing these isolation cuts as a function of the $$R_{\text {iso}}$$ parameter are shown in Fig. 4. For both analyses, the *W*-had background falls off considerably faster than the signal as $$R_{\text {iso}}$$ is increased. Both backgrounds fall so quickly in the $$\pi \pi \pi $$ case that there is essentially no background in this MC study for $$R_{\mathrm{iso}}\ge 0.06$$. The *W*-tau background does not fall off for large values of $$R_{\mathrm{iso}}$$ in the $$\pi \gamma $$ analysis. This is caused by events where the tau decays $$\tau ^+ \rightarrow \pi ^+ \bar{\nu }_\tau $$ ($$\sim $$$$ 10\,\%$$ of the time) to create the single isolated pion, and where there is a hard, well-separated photon radiated from elsewhere in the event (top, *b*-quark, initial state, electron). Since the event is relatively empty (with three neutrinos), the cost of this is $$\sim $$$$\alpha _{EM}$$, and so this background tracks the signal, being below it by a factor of $$\sim $$$$10^{-1}\times 10^{-3}$$. It is reducible to the extent to which the pion can be identified as coming from a tau decay, using displaced vertex tagging, although given that the tau will be well boosted in the laboratory frame we do not expect this to provide any significant experimental improvement.^6^ The $$\pi \pi \pi $$ signal is seen to be less efficient than the $$\pi \gamma $$, simply because in this case three particles have to pass both transverse momentum and isolation cuts. We plot the shape of the background $$M_{\pi \gamma }$$ distributions in the $$\pi \gamma $$ analysis in Fig. 5. The peak of the distributions is driven by the $$p_T$$ cut on the jets, and lowering this cut moves the peak away from the signal region, which would make a polynomial fit of the background shape more reliable. However, the gain in the number of background events passing the cuts acts in the opposite direction, and after studying a $$p_T$$ cut of 20 GeV, it appears marginal as to whether one would want to lower the $$p_T$$ cuts to try and gain sensitivity. The reconstructed *W* mass from the signal samples is plotted in Fig. 6, for values of $$R_{\mathrm{iso}}$$ used in the below analysis.

We now turn to discussing the observation prospects of these decays at the HL-LHC, with 3ab$$^{-1}$$ of data. Of order $$10^9$$$$t\bar{t}\rightarrow W^\pm l^\mp \nu _l b \bar{b}$$ events passing the $$t\bar{t}$$ selection cuts are expected, where *l* is an electron or a muon. The number of $$t\bar{t}\rightarrow W^\pm l^\mp \nu _l b \bar{b}$$ events must be multiplied by the branching ratio of $$W\rightarrow $$ hadrons and $$W\rightarrow \tau \nu _\tau $$ to obtain the number of *W*-had and *W*-tau events, and by the branching ratio of $$W\rightarrow \pi \gamma $$ and $$W\rightarrow \pi \pi \pi $$ to obtain the number of signal events in each decay mode. We use the approximation that the analysis for the negatively charged $$W^-$$ signal decay simply gives a factor two in statistics and, given the difficultly in obtaining events at larger values of $$R_{\mathrm{iso}}$$, we assume the $$R=0.06$$ background shape for both backgrounds in the $$\pi \gamma $$ analysis (we find their shapes remain reasonably stable as $$R_{\text {iso}}$$ is increased – see Fig. 5). For $$W\rightarrow \pi \gamma $$, given that the number of *W*s passing the $$t\bar{t}$$ acceptance cuts is of order $$\sim $$$$10^{9}$$, and calculations of the standard model branching ratio are in the region $$\sim $$$$10^{-9}$$–$$10^{-6}$$, clearly the HL-LHC could only possibly have sensitivity to this decay in the upper region of this window. Given the spread in theoretical predictions, and to give an idea of the level of exclusion limit the HL-LHC could set, we estimate the value of branching ratio which would allow for a $$3\sigma $$ signal, estimating the significance with $$N_{\text {sig}}/\sqrt{N_{\text {bkg}}}$$, where $$N_{\text {sig}}$$, $$N_{\text {bkg}}$$ are the number of signal and background events in the region 78–84 GeV of the $$M_{\pi \gamma }$$ distribution. Optimising over $$R_{\mathrm{iso}}$$ we find that for a value of $$R_{\mathrm{iso}}=0.15$$, a 3$$\sigma $$ discovery is obtained for a branching ratio of $$6\times 10^{-7}$$, where $$N_{\text {sig}}=70$$ and $$N_{\text {bkg}}\simeq 450$$. The number of events in the tail above the region 78–84 GeV is $$\sim $$$$3000$$, meaning that the statistical error considered above dominates. This assumes no displaced vertex tagging applied to the *W*-tau background but, given the one-particle decay mode of the tau, this is presumably reasonable. Even though this branching ratio is still above the likely standard model prediction, exclusion limits could be set lower than the current best limits set by CDF. For $$W\rightarrow \pi \pi \pi $$ the entire suppression of background for $$R_{\mathrm{iso}}\ge 0.06$$ found in the three-pion decay channel means that a discovery estimate can be translated directly: a sensitivity to a branching ratio of a few$$\times $$$$10^{-7}$$, which probes well inside the expected standard model region.

For this analysis we have used leading order event generation, and made no estimate of the theoretical uncertainties in doing so, although as the analysis is shape driven we do not expect NLO QCD effects to have a large effect on this. We also do not take into account any of the realistic collider effects, in particular the problem of pileup and detector effects. A full study of these effects is beyond the scope of this note, but we point out some important handles and improvements which can be made in the analyses which we hope will ameliorate the inevitable degradation of the results presented here. Firstly, important information is contained in the direction of the three-momentum of the particles – particularly well measured for the charged pions – since these tracks should point back to the interaction vertex (flagged by the lepton in the $t\bar{t}$ event) coming as they do directly from the *W* decay. This can be used to kill background coming from secondary isolated pion production (such as tau decay) and to help deal with pileup contamination – the vertex must be the same as that determined for the $t\bar{t}$ event. Whether this alone will be enough to control pileup and whether new experimental techniques can be invented to increase sensitivity to exclusive hadronic decays under LHC pileup conditions remain questions to be answered by a detailed study. Secondly, given that the background determination will be data driven, a useful observation is that the sign of the lepton coming from the tagging side of the $t\bar{t}$ event fixes the sum of the charges of the signal decay products. Events passing the single particle cuts with the wrong charge sum provide important information on the nature of the background, even in the signal region. Thirdly, the mass of the single particle jets and one of the *b*-jets can be required to be in the vicinity of the top mass, which will further suppress background. Finally, electromagnetic calorimeter profiling can be used to tighten the definition of ‘single $\gamma$’-jets above by discriminating between true single, hard photons and multiple photons (for example, as is done in $h\rightarrow \gamma \gamma$ analyses to suppress $\pi ^0\rightarrow \gamma \gamma$ contamination).

The above analysis carries over directly to two- and three-particle decays where charged pions are replaced by charged kaons or (anti-)protons, since these too are stable as far as the detector is concerned. In reality, particle identification (PI) is done on a statistical basis, so these measurements would overlap into each other. Similar to the wrong charge sum exploration of the background, groups of decay products are forbidden, for example $W^+\rightarrow p \pi ^+ \pi ^-$, although this has to be convoluted with the uncertainty in PI described above. Three-particle *W* decay to charmed mesons, e.g. $W^+\rightarrow \{D^+,D^+_s,\ldots \} \pi ^+\pi ^-$, and a whole spectrum of higher spin mesons and baryons could be envisaged, but unlike the pions, kaons and protons, these particles decay before reaching the detector. This could give rise to distinctive signatures, and it would be interesting to investigate them, in particular the details of the complications arising from neutrinos in the decays, and the way in which tagging techniques would fit with the isolation techniques used here. For example, jet substructure techniques can look for particular decay patterns inside the cone of size $R_{\mathrm{iso}}$ and not make the single particle veto if a match is found. However, as the results here indicate that observation in the simpler ‘stable’ hadron modes will be challenging, we think it unlikely that such measurements would be feasible given the additional experimental difficulties they entail.

We briefly comment on the possibilities of precision mass measurement of the *W* boson in the three-pion decay channel, since a fully visible final state makes it possible to directly construct a mass peak, in contrast to the usual techniques that have to deal with missing energy originating from a neutrino in a leptonic decay channel. The current uncertainty on the *W* mass is $15\,$MeV [38], and the most precise determination is obtained by fitting the transverse mass distribution in the lepton-neutrino decay channel at the Tevatron. The mass resolution here should be very good, with the average $p_T$ of the hardest positively charged pion $\langle p_T\rangle \sim 60\,$GeV, and the $p_T$ of the two other pions sharply peaked toward the cutoff $p^{\mathrm{jet}}_{T~\mathrm{min}}=25\,$GeV, with an average $\langle p_T\rangle \sim 40\,$GeV. However, the statistical uncertainty scales like $\Delta M \sim \Gamma _W/\sqrt{N_{\mathrm{sig}}}$, where $\Gamma _W=2.085\,$GeV is the width of the *W*, and so, to obtain a competitive level of precision, of order $10^4$ events are necessary, beyond the reach of the HL-LHC, even with the most optimistic branching ratio of $10^{-5}$. It would be possible to investigate the use of higher multiplicity exclusive particle final states, which have substantially larger branching ratios, but this increases the chances of these particles falling out of the detector geometry, or falling into the *b*-jets and other QCD jets.

## Conclusions and outlook

Top quark pair production provides a potential high-statistics environment for studying the properties of *W* boson decays with a limited trigger bias. Triggering on two *b*-jets and the leptonic decay of one *W* suppresses QCD backgrounds to both the trigger and the analyses, and requiring (transverse) top mass reconstruction, QCD backgrounds can be even further reduced. Around $10^9$ additional *W*s on the other side of the event will be produced in this way after the HL-LHC run. In this note we have discussed making measurements of exclusive hadronic decays of the *W* bosons in this environment. We showed that, by using isolation cuts embodied by single particle jets, it is possible that the LHC reaches the sensitivity required for measuring what would be the first exclusive hadronic decay of a fundamental standard model boson. We considered as an explicit example the decays $W^+\rightarrow \pi ^+\gamma$ and $W^+\rightarrow \pi ^+\pi ^+\pi ^-$, and concluded that while the two-particle decay has a branching ratio which is likely too small for observation, the three-particle decay has potential to be measured after the HL-LHC run.

However, a detailed and realistic experimental simulation is necessary to determine whether the conclusions presented in this note are robust. We have pointed out further experimental handles that can be used to improve aspects of the analysis. It will be interesting to see whether single particle jet isolation, used here primarily for its simplicity, is a useful technique after detector effects and pileup are taken into account. We expect that more usual isolation criteria (such as requiring hadronic activity of less than some energy in a cone around a particle, similar to those already employed in the $h \rightarrow \gamma \gamma$ analyses and in tau-lepton identification) can be equally well employed to search for these exclusive hadronic states. It will also be interesting to see if isolation requirements can be useful in more hostile environments, and investigate further scenarios where a trade-off in high luminosity in favour of isolated signals is beneficial in extracting new measurements.

Although semi-exclusive hadronic measurements of the form $W\rightarrow P X$ (where *P* is a named particle, and *X* is anything) are very challenging in generic *W* production at a hadron collider, these decays also have potential to be studied in the $t\bar{t}$ environment. A natural extension of this work would be to investigate the degree to which a *W* semi-exclusive decay table, akin to the entries in the *Z* decay table, could be built up during the course of the HL-LHC run. This would again exploit the fact that after the $t\bar{t}$ tagging procedure a *W* boson remains in the event, to which one could assign particles $P=J/\psi , D^{\pm },B_S^0$, etc, if they are subsequently observed. In principle, such measurements could be easier than the fully exclusive decays studied here due to their considerably larger branching ratios.

Experimental observation of an exclusive hadronic decay mode of the *W* at a hadron collider would bolster proposals in the literature to search for exclusive Higgs decays at these machines. It is tempting to speculate on the implications of this study for such measurements, in particular the preference for three-body decay modes, due to both the increased branching ratio and the background reduction seen here. However, the decay mechanism is different enough to warrant further study in this direction and, more importantly, the triggering requirements for dealing with the collider background are very different between what we study here and the case of Higgs production.^7^ We leave such considerations to future work. It is clear, however, that if experimental techniques were honed so as to measure an exclusive decay of the *W*, this would be invaluable in assessing future exclusive Higgs decay prospects.

The branching ratios for the exclusive hadronic decays considered here are pushing the limits of the statistics available at the LHC. At a future hadron collider, such as a 100 TeV *pp* collider, up to two orders of magnitude more $$t\bar{t}$$ events are expected. The details of the detectors and experimental methods for dealing with pileup and hugely energetic particles are completely open ended. However, the bulk of the $$\sim $$$$10^{12}$$$$t\bar{t}$$ events will be produced close to threshold, such that the dynamics of the events themselves will be very similar to LHC events. Furthermore, backgrounds which are not *gg* initiated will not grow as fast as the $$t\bar{t}$$ cross section. It seems justified to assume that the reach of such a machine can be estimated by scaling with the additional luminosity the results obtained here, accessing the region of the two-particle $$W\rightarrow \pi \gamma $$ and related decays. As a very rough estimate, repeating the analysis with $$10^{11}$$*W*s, a 3$$\sigma $$ observation of $$W\rightarrow \pi \gamma $$ with a branching ratio of $$6\times 10^{-8}$$ is found, and a $$W\rightarrow \pi \pi \pi $$ decay with a branching ratio $$\sim $$$$10^{-7}$$ should yield a few thousand events. These numbers could in principle compete with the reach of the proposed future circular electron–positron colliders, which should collect clean samples of $$O(10^8)$$ W bosons.

A rich amount of possibilities for extending the known *W* decay table lies in exclusive hadronic decays alone. But, akin to the entries in the *Z* decay table, this can be bolstered further already at the LHC, through searches in the $t\bar{t}$ environment for lepton flavour and number violating *W* decays,^8^ and improvements in the precision of the branching ratios to leptons. Finally, *W* boson properties are just one aspect of the utilisation of the very high statistics in a $t\bar{t}$ environment. Because roughly a ninth of *W*s decay into taus, and another third decay into charmed hadrons, a similar number of these particles as *W*s opens up the possibility of a detailed study of their properties in turn. Furthermore, as discussed in Ref. [39], the *b* quarks produced in the top decay create an enormous number of *B*-hadrons, which can have their *b* or $\bar{b}$ nature determined via the sign of the lepton from the decay of the associated *W*, after (transverse) top mass reconstruction. Looking to the far future, there is a very open playing field as to the details of new hadron colliders, with plenty of room for innovative searches and detectors. In this context, we look forwards to further work as regards the question: can huge statistics in the bush compete with a smaller number of clean events in the hand?

## Figures and Tables

**Fig. 7:**
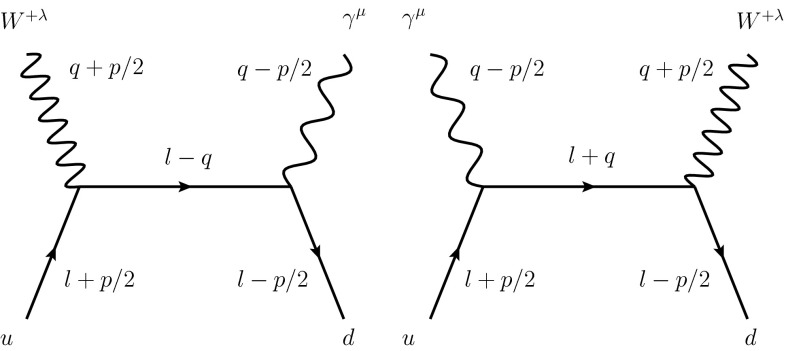
Leading order in QCD diagrams for the OPE calculation of the contribution to the decay $W\rightarrow \pi \gamma$ shown in Fig. 3b

## Appendix A: OPE for the decay rate $W\rightarrow \pi \gamma$

Here we review Manohar’s calculation of $Z\rightarrow W\pi$, Ref. [15], simply making the replacement of *Z* with $\gamma$ (and using crossing symmetry) to apply it to the process of interest here, and we compare with the AMP result.

The contribution to Fig. 3b at leading order in QCD involves a calculation of

A1

using the kinematics shown in Fig. 7. This is expanded in a power series in the variable $\omega = -2 p\cdot q/q^2$, and only the leading twist two operators are retained, defined as

A2 $$\begin{aligned} O_L^{\mu _1\ldots \mu _n} = \text {Sym}\left[ (i/2)^{n-1} \bar{d} \gamma ^{\mu _1} \overset{\leftrightarrow }{D}\,^{\mu _2}\ldots \overset{\leftrightarrow }{D}\,^{\mu _n}P_L u\right] \nonumber \\ \end{aligned}$$

where $\text {Sym}[. .]$ symmetrises the expression with respect to the Lorentz indices. Defining the matrix elements of these operators between the pion and vacuum as

A3 $$\begin{aligned} \langle \pi (p) | O_L^{\mu _1\dots \mu _n} | 0 \rangle \equiv a_n\,f_\pi p^{\mu _1}\ldots p^{\mu _n} \end{aligned}$$

we have $a_1=1/\sqrt{2}$ and all $a_n=0$ for *n* even.^9^ For a real photon of momentum $k=q-p/2$ and a real *W* of momentum $p_W=p+k=q+p/2$, the polarisation vectors satisfy

A4 $$\begin{aligned}&\epsilon \,(k) \cdot k = 0 ,\end{aligned}$$

A5 $$\begin{aligned}&\epsilon \,(p_W) \cdot p_W =0 . \end{aligned}$$

This OPE determines the contribution Fig. 3b to be

A6 $$\begin{aligned}&A_{\text {3(b)}}= V_{ud} \frac{i g}{\sqrt{2}}(-i e)\epsilon _{\mu }(k)\epsilon _{\lambda }(p+k) f_\pi \left( \frac{ a_1g^{\mu \lambda }\,p\cdot q}{q^2} \right. \nonumber \\&\quad +\left. ( p\cdot k\,g^{\mu \lambda } - p^\mu k^\lambda ) \,\frac{1}{q^2}\,\sum _{n=2}^{\infty } [Q_u+(-1)^nQ_d] a_n \omega ^{n-1} \right. \nonumber \\&\quad -\left. i \epsilon ^{\mu \lambda \rho \sigma } p^\rho k^\sigma \,\frac{1}{q^2}\,\sum _{n=1}^{\infty } [Q_u-(-1)^nQ_d] a_n \omega ^{n-1} \right) . \end{aligned}$$

Returning to the contribution from Fig. 3a, $A_{\text {3(a)}}$, we have

A7 $$\begin{aligned} A_{\text {3(a)}} = V_{ud} \frac{i g}{\sqrt{2}}(-i e)\epsilon _{\mu }(k)\epsilon _{\lambda }(p+k) \frac{f_\pi }{\sqrt{2}} \left( \frac{2 g^{\mu \lambda } \,p\cdot q }{p^2-m_W^2 } \right) .\nonumber \\ \end{aligned}$$

The first term in Eq. A6 cancels with Eq. A7 in the full amplitude, and we find agreement with the general Lorentz structure as in AMP,

A8 $$\begin{aligned}&A_{\text {3(a)}}+A_{\text {3(b)}} = \frac{-e g V_{ud}}{\sqrt{2}} \epsilon _\mu (k)\epsilon _\lambda (p+k) \nonumber \\&\quad \!\times \! \left[ \mathcal {A}_\pi (m_W^2) (p\cdot k\, g^{\mu \lambda } {-} p^\mu k^\lambda ) \!+\! \mathcal {V}_\pi (m_W^2) i \epsilon ^{\mu \lambda \rho \sigma } p_\rho k_\sigma \right] \nonumber \\ \end{aligned}$$

with our expressions for the axial- and vector-like form factors $\mathcal {A}_\pi$ and $\mathcal {V}_\pi$ can be read off from Eq. A6. The expansion parameter

A9 $$\begin{aligned} \omega =\frac{ 2(m_\gamma ^2-m_W^2)}{(m_\gamma ^2+m_W^2)}=-2, \end{aligned}$$

and so using the first term in this series can only be seen as giving an order of magnitude estimate for the decay rate – higher order corrections are clearly important. The situation is analogous to that of the calculation of $Z\rightarrow \pi \gamma$ in [15], for which the expansion parameter takes the same value. Taking just the leading term, the spin averaged rate obtained is

A10 $$\begin{aligned} \Gamma (W\rightarrow \pi \gamma ) = \frac{\pi \alpha ^2 |V_{ud}|^2 f_\pi ^2}{54 m_W \sin \theta _W^2} \sim 10^{-9}\, \text {GeV}. \end{aligned}$$

This is smaller than the AMP result by a factor of 2/9. AMP argued $|\mathcal {A}_\pi (s)/ \mathcal {V}_\pi (s) | \underset{s\rightarrow \infty }{\rightarrow } 1$, taking the form of the vector-like form factor from the BL asymptotic limit $\mathcal {V}_\pi (s) \underset{s\rightarrow \infty }{\rightarrow } - \sqrt{2}f_\pi /s$. Since the decay rate is proportional to $| \mathcal {V}_\pi |^2 (1+ |\mathcal {A}_\pi / \mathcal {V}_\pi |^2 )$ a factor of 1/2 arises here, since in the above we have in the leading term $\mathcal {A}_\pi =0$. A further factor of 2/3 in the amplitude provides a further factor of 4/9 in the decay rate. A factor of 2/3 difference with the BL formula (as used by AMP), which is found by taking the leading term in the approach above, was noted by Manohar [15]; taking this into account, the two approaches give consistent results.

## Footnotes

1 The CDF experiment improves on the limit quoted in the current PDG by an order of magnitude to $$<$$$$6.4\times 10^{-6}$$ [2].

2 Note added: after the release of a preprint of this paper, a publication by other authors [16] has presented this calculation (and many other exclusive modes) using the light cone distribution amplitude method, including higher order terms and with a detailed study of systematic errors, leading to a branching ratio of $(4.00\pm 1.0) \times 10^{-9}$ (all quoted errors combined in quadrature). The order of magnitude estimate that we presented here is consistent with this more rigorous evaluation.

3 This asymptotic limit for $\gamma ^\star \gamma \pi$ is defined when the mass of the off-shell photon $Q^2\rightarrow \infty$. However, it too receives important higher order terms when $Q^2$ is very large but finite [15]. This form factor is relevant for the off-shell $\gamma ^\star \gamma \rightarrow \pi$ production anomaly at Belle [19] and BaBar [20]; see e.g. [21–29].

4 Such effects are suppressed since they can only be observed through a correlation in the *W* spin with the spin of another particle in the event, leading, if present, to possible correlations in the momenta of the *W* decay products with e.g. the lepton or *b*-jet on the other side of the event. Given the tiny rates, we believe such effects will not be measurable. However, for completeness we mention that the $W\rightarrow \pi \gamma$ decay should proceed $\propto (1+\cos ^2\theta )$, as only transverse polarisations of the *W* and the $\gamma$ contribute to the decay. For the $W\rightarrow 3\pi$ decay, a more involved Dalitz-style plot should be analysed, using a similar analysis to e.g. [35].

5 A check was put in place to disregard any of these events where the $W^+$ decays into a two- or three-particle state after hadronisation.

6 The analogue of this background does not affect the $\pi \pi \pi$ analysis, since the boost of the $\tau$ means the decay $\tau \rightarrow \pi \pi \pi \nu$ is pencil-like.

7 Of course $t\bar{t}H$ production would have similar triggering requirements, but the cross section is around three orders of magnitude smaller than $t\bar{t}$ production, reducing sensitivity to rare branching ratios.

8 For example, see e.g. [5] for a study of the parameter space of heavy Majorana neutrinos which can be accessed through on-shell *W* decay.
